# Selection of Reference Genes for RT-qPCR Analysis Under Intrinsic Conditions in the Hawthorn Spider Mite, *Amphitetranychus viennensis* (Acarina: Tetranychidae)

**DOI:** 10.3389/fphys.2019.01427

**Published:** 2019-11-19

**Authors:** Jing Yang, Yue Gao, Zhongfang Liu, Junjiao Lu, Yuying Zhang, Pengjiu Zhang, Jianbin Fan, Xuguo Zhou, Renjun Fan

**Affiliations:** ^1^Shanxi Key Laboratory of Integrated Pest Management in Agriculture (IPMA), Institute of Plant Protection, Shanxi Academy of Agricultural Sciences, Taiyuan, China; ^2^Department of Entomology, University of Kentucky, Lexington, KY, United States

**Keywords:** RT-qPCR, reference genes, housekeeping genes, hawthorn spider mites, *Amphitetranychus viennensis*, cell-content feeding

## Abstract

Hawthorn spider mite, *Amphitetranychus viennensis* Zacher, is one of the most devastating pests of deciduous fruit trees. The overall goal of this research is to develop a standardized protocol for real-time quantitative reverse transcription PCR (RT-qPCR) analysis in *A. viennensis* following the MIQE (minimum information for publication of Quantitative real time PCR experiments) guidelines. Based on the previous knowledge, we hypothesized that internal references for RT-qPCR analysis reside in housekeeping genes (HKGs). To test this hypothesis, we examined the stability of nine HKGs from *A. viennensis*, including *18S ribosomal RNA* (*18S*), *28S ribosomal RNA* (*28S*), *Elongation factor 1-alpha* (*EF1A*), *Actin3*, *V-ATP vacuolar-type H^+^-ATPase* (*V-ATPase*), α*-tubulin* (α*-tubulin*), *Ribosomal protein L13* (*RPL13*), *40S ribosomal protein S9* (*RPS9*), and *Glyceraldehyde 3-phosphate dehydrogenase* (*GAPDH*). The expression profile of these candidates under intrinsic conditions was evaluated by a panel of computational programs, including geNorm, Normfinder, BestKeeper, and ΔCt method. Based on RefFinder, a comprehensive software integrating all four above-mentioned algorithms, *V-ATPase*, *Actin3*, and *GAPDH* are the top three reference genes, which are stably expressed across all the intrinsic conditions, including developmental stage, sex, and diapause. In addition, we compared reference genes recommended for different developmental stages among the nine cell-content feeding arthropods, including four spider mites, *A. viennensis, Tetranychus urticae, Tetranychus cinnabarinus*, and *Panonychus citri*, and five hemipterans, *Myzus persicae, Aphis gossypii, Toxoptera citricida, Lipaphis erysimi*, and *Sogatella furcifera*. Not surprisingly, rRNAs and ribosomal proteins, the most abundant RNA species, is the top choice, and follows by *EF1A, Actin, GAPDH*, and *tubulin.* Information present here lays the foundation for the genomic and functional genomic research in cell-content feeding arthropods in general and *A. viennensis* in particular.

## Introduction

Analyzing differential gene expression in various biosynthesis pathways is an integral part of the genomics research (Ginzinger, 2002; Van Guilder et al., 2008). Because of its specificity, reproducibility, and sensitivity, real-time quantitative reverse transcription PCR (RT-qPCR) is considered by many as one of the best methods to quantify gene expression (Valasek and Repa, 2005; Park et al., 2008). Limitations, however, still exist, including variation in RNA quantity, reverse transcription normalization, PCR amplification efficiency and experimental procedures between samples (Bustin, 2002; Bustin et al., 2005; Strube et al., 2008). To ensure reliability and accuracy, one critical step in RT-qPCR analysis is to use reference genes as internal controls from the same samples (Vandesompele et al., 2002). Housekeeping genes (HKGs), which are constitutively expressed and maintain the basic cellular functions in the cell, have been used extensively as internal references for RT-qPCR analysis (Yang et al., 2015b, 2018). Ideally, reference genes should be stably expressed across different biotic and abiotic conditions. However, we have not yet identify a reference gene can be stably expressed across any given experimental conditions. Therefore, a systematic and customized study of reference genes for each tested species is critically important (Gutierrez et al., 2008; Hruz et al., 2011; Kozera and Rapacz, 2013).

The hawthorn spider mite, *Amphitetranychus viennensis* Zacher, is a devastating pest on deciduous fruit trees. *A. viennensis* is broadly distributed in Asia and Europe (Ehara and Gotoh, 1990; Migeon et al., 2010). *A. viennensis* causes yellow spots on the leaves by sucking, and it also causes indirect damages by laying eggs on the leaf surface and covering the individual egg with silky threads. The infestation by *A. viennensis* can lead to either curled-up leaves and/or defoliation, which ultimately reduces the photosynthetic capability of plants (Kafil et al., 2007; Li et al., 2007). The life cycle of *A. viennensis* typically consists of four stages: egg, larva, nymph and adult, in which nymphal stage has two instars, protonymphs, and deutonymphs (Kasap, 2003). *A. viennensis* has a haplodiploid sex determination system. Diploid offspring develops from fertilized eggs, and is typically female, while haploid offspring develops into male from unfertilized eggs. Under adverse conditions, such as long night, low temperature, malnutrition, and predation risks, *A. viennensis* will transition into a diapause stage with an array of physiological, morphological, and molecular modifications, similar to other spider mites (Bryon et al., 2017).

The management of *A. viennensis* has been primarily relied on the synthetic chemicals (Li et al., 2007). As a result, *A. viennensis* has developed resistance to almost all the commercially available acaricides (Li et al., 2007). The development of pesticide resistance, combined with pesticide residues in both food products and the environment, has led to the search for alternative pest management strategies, including the emerging biotechnology of RNAi (Suzuki et al., 2017; Yoon et al., 2018). However, the lack of genomic resources and companion toolsets in *A. viennensis* becomes a limiting factor for such efforts. To expand *A. viennensis* research into the Genomics Era and to facilitate the adoption of RNAi into the existing integrated pest management strategies, we have sequenced the genome and transcriptomes of *A. viennensis* (JY unpublished data).

To take full advantage of the newly established genomic resources, we first would like to develop a standardize RT-qPCR procedure in *A. viennensis* following the MIQE (minimum information for publication of quantitative real time PCR experiments) guidelines (Bustin et al., 2009). A standardized RT-qPCR analysis is instrumental for the subsequent genomic and functional-genomic research. Our overarching hypothesis is that the internal references for RT-qPCR analysis in *A. viennensis* can be selected from the HKGs. To test this hypothesis, we examined the stability of nine HKGs identified from *A. viennensis* transcriptome under different experimental conditions. These candidates, including *18S ribosomal RNA*(*18S*), *28S ribosomal RNA* (*28S*), *Elongation factor 1-alpha* (*EF1A*), *Actin3*, *V-ATP vacuolar-type H^+^-ATPase* (*V-ATPase*), α*-tubulin* (α*-tubulin*), *Ribosomal protein L13* (*RPL13*), *40S ribosomal protein S9* (*RPS9*), and *Glyceraldehyde 3-phosphate dehydrogenase* (*GAPDH*), have been used extensively as the internal references for RT-qPCR analysis (Sun et al., 2010; Niu et al., 2012; Pan et al., 2015; Shang et al., 2015; Yang et al., 2015a, 2018; Koramutla et al., 2016; Ma et al., 2016; Kang et al., 2017; Tan et al., 2017; Lü et al., 2018). The expression of these candidates under various intrinsic conditions, including developmental stage, sex, and diapause, was evaluated by a panel of computational programs, including geNorm, Normfinder, BestKeeper, and ΔCt method. Finally, a specific set of reference genes is recommended for each intrinsic conditions, respectively, based on RefFinder, a comprehensive analytical software integrating all four above-mentioned algorithms (Xie et al., 2012).

## Materials and Methods

### *Amphitetranychus viennensis* Colony Maintenance

*Amphitetranychus viennensis* (Acari: Tetranychidae) adult females were collected from crabapple *Malus* “Radiant,” at Shandong Agricultural University in August 2017. Larvae and adults were maintained in the laboratory and provisioned with peach leaves (*Prunus davidiana*) at 26 ± 0.5°C, 16L: 8D photoperiod, and 50% relative humidity. The identity of *A. viennensis* was confirmed by a combination of morphological traits (Ehara and Gotoh, 1990; Navajas et al., 1997) and a mitochondrial DNA marker, COI. The 728bp COI fragment was amplified by a pair of conserved primers (Av-COI-F1: TTGGAGGATTTGGAAATTGA and Av-COI-R: TCCTGTTGGAATGGCAATAA). Based on NCBI BLAST search, our COI fragment has a sequence similarity >99% with *A. viennensis* mitochondrial genome (GenBank: KX886344).

Developmental stage, sex, and diapause of *A. viennensis* were determined by the morphological traits using a light microscope (SMZ171, Motic, Xiamen, China). The image of *A. viennensis* at different life stages was recorded using a Research Stereo Microscope (SMZ18, Nikon, Tokyo, Japan).

### Intrinsic Conditions

The developmental stage includes egg (*N*≈2000), larvae (*N*≈2000), protonymph (*N*≈800), and deutonymph (*N*≈400). Sex of adults including male (*N*≈400) and female (*N*≈100). Diapause stage of adult females includes the non-diapause (*N*≈100), pre-diapause (females begin to enter into the diapause stage but not yet complete the process, *N*≈100), and diapause female (*N*≈100). Samples were preserved in 1.5 ml centrifuge tubes and snap frozen immediately in liquid nitrogen before storage at −80°C. Each sample collection/experiment was replicated three times independently.

### Total RNA Extraction and cDNA Synthesis

Total RNA was extracted using TRIzol reagent (Reagent Catalog NO. 15596026, Ambion, CA, United States) following the manufacturer’s instruction with minor modification. Each sample was ground in liquid nitrogen using a 2 ml glass grinder and then homogenized in 1 ml TRIzol reagent. The homogenized samples were centrifuged for 10 min at 12,000 × *g* at 4°C. The clear supernatant was transferred to a new tube, and then incubated for 5 min to completely dissociate nucleoproteins complex. 0.2 ml of chloroform was added to the supernatant, then the centrifuge tube was capped tightly and shaken vigorously by hand for 15 s. The sample was incubated at room temperature for 2–3 min and centrifuged for 15 min at 12,000 × *g* at 4°C. The upper aqueous phase containing the RNA was transferred to a new tube. 0.5 ml of isopropanol was added to the aqueous phase and incubated at room temperature for 10 min. Then, the sample was centrifuge for 10 min at 12,000 × *g* at 4°C. The supernatant was discarded with a micropipettor and the white pellet at the bottom of the tube was resuspend in 1 ml ethanol. The sample was vortexed briefly and centrifuged for 5 min at 7,500 × *g* at 4°C. The supernatant was discarded, the RNA pellet was dried in air for 5–10 min. The extracted RNA was resuspended in 30 μl of nuclease-free water. The concentration of RNA was quantified with a NanoDrop NC2000.

Single-stranded cDNA was synthesized for each biological sample from total RNA using the PrimeScript RT reagent Kit with gDNA Eraser (Perfect Real Time) (Code No. RR047A, Takara, Dalian, China). The first step is to remove genome DNA, and the reaction contains 2.0 μl of 5 × Eraser buffer, 1.0 μl gDNA Eraser, 1.0 μg of total RNA, and RNase free dH_2_O was added up to 10 μl. The reaction system was incubated at 42°C for 2 min, then store at 4°C. The second step is a reverse transcription reaction, which contains 10 μl of first step reaction mixture, 1.0 μl of PrimeScript RT Enzyme Mix I, 4.0 μl of RT Primer Mix, 4.0 μl 5× PrimerScript Buffer 2 (for Real Time), 1.0 μl of RNAase-free ddH_2_O. The reaction system program included 37°C for 15 min, 85°C for 5 s. The cDNA was stored at −20°C and diluted 10× for the subsequent RT-qPCR studies.

### Candidate Reference Genes and Primer Design

All of the nine HKGs that are commonly used in RT-qPCR analysis were selected as the candidate (Table 1). Majority of these genes have been previously used as the reference genes in other spider mite species and insect (Lü et al., 2018). The candidate genes were selected from GenBank and the transcriptome of *A. viennensis* which sequenced by Personal Biotechnology Co (Shanghai, China), including *18S* (GenBank Accession Number: AB926293.1), *28S* (KU323547.1), *EF1A* (MN603410), *Actin3* (MN603409), *V-ATPase* (MN603411), α*-tubulin* (MN603413), *RPL13* (MN603414), *RPS9* (MN603415), and *GAPDH* (MN603412). Primers for the RT-qPCR analysis were designed by Primer3Plus online^1^ (Untergasser et al., 2007). PCR was performed in 50 μl reactions containing GoTaq Green Master Mix (Reagent Catalog NO. M7122, Promega, Madison, WI, United States) 25 μl, 1.5 μl of each primer (10 μM each) and 50 ng first-strand cDNA. The PCR parameters were as follows: one cycle of 94°C for 3 min; 35 cycles of 94°C for 30 s, 55°C for 1 min and 72°C for 1 min; a final cycle of 72°C for 10 min. Reactions were performed in a C1000 thermalcycler (BioRad). PCR products were separated on appropriate agarose gels, corresponding bands were extracted and purified from the gels, and the purified DNA fragments were cloned into the Peasy-T1 vector (Reagent Catalog NO. CT101, Trangen, Beijing, China) sequencing confirmation.

**TABLE 1:** Primer sets used in this study.

**Gene name**	**Abbreviation**	**GenBank accession No.**	**Primers (5′-3′)**	**Length (bp)**	**Tm (°C)**	**Efficiency (%)**	***R*^2^**	**Slope**	***y*-intercept**
**Candidate reference gene**									
*18S ribosomal RNA*	*18S*	AB926293.1	F: CCGCCCTAGTTCTAACCATAAA R: GTTTCAGCTTTGCAACCATACT	132	82.0	102.4	0.98	−3.27	33.63
*Elongation factor 1-alpha*	*EF1A*	MN603410	F: AGGGTTCCAAATTGGAAGGTAAA R: GTGGAAGTCGAAGAGCCTTGT	93	80.5	99.1	1.00	−3.34	33.98
*28S ribosomal RNA*	*28S*	KU323547.1	F: AGCTAAGACCCCTTGGCAAC R: TAAGGATAGGGGGCCTTCCC	132	80.5	99.3	0.99	−3.35	31.82
*Actin3*	*Actin3*	MN603409	F: CCAAGGAGTAATGGTCGGTATG R: CCATGCTCAATTGGGTATTTAAGG	102	79.1	90.3	0.99	−3.58	33.29
*Vacuolar-type H* + *-ATPase*	*V-ATPase*	MN603411	F: GTCGTGGTTTCCCAGGTTAT R: CGTTTGGCATGGTAAGAATGG	117	81.0	93.6	0.98	−3.48	30.42
*Glyceraldehyde 3-phosphate dehydrogenase*	*GAPDH*	MN603412	F: TGATGCACCCATGTTTGTAAT R: GGGGCAAGACAGTTAGTGGTA	99	78.5	90.6	0.99	−3.57	31.93
α*-tubulin*	α*-tubulin*	MN603413	F: ATTCGTTGACTGGTGCCCAA R: TGCCCGTTGAACTTTGGCTA	97	80.0	95.0	0.98	−3.48	30.42
*Ribosomal protein L13*	*RPL13*	MN603414	F: CGCCCTTATAGCCTCTTCTTATT R: CCACTACAACCCTTTGTCCTT	102	77.9	92.4	1.00	−3.52	40.73
*40S ribosomal protein S9*	*RPS9*	MN603415	F: CTGCTCGAGAGTTGCTGACT R: AGACCCAAGACGTAATCGAGC	133	78.0	106.5	0.99	−3.18	39.49
**Target gene**									
*CREB-binding protein*	*CBP*	MN607216	F: CAGCGCCACAATCTAATCAA R: TTGACCACCGGTACCATTTT	140	76.5	93.1	1.00	−3.50	43.70

### Real-Time Quantitative Reverse Transcription PCR (RT-qPCR) Analysis

Gene-specific primers (Table 1) were used in RT-qPCR reactions (20 μl), containing 6.8 μl of ddH_2_O, 10 μl of 2 × TB Green Premix Taq II (Tli RNaseH Plus) (Code No. RR820A, Takara, Dalian, China), 0.8 μl of each specific primer (10 μM), and 1.6 μl of first-strand cDNA template. The RT-qPCR program included an initial denaturation for 3 min at 95°C, 40 cycles of denaturation at 95°C for 5 s, annealing for 30 s at 55°C, and extension for 30 s at 72°C, followed by melt curve analysis using default parameters by steady increase in temperature from 55°C to 90°C. Reactions were performed in a CFX96 Real-Time PCR Detection System (BioRad). The presence of a single peak in the melting curve analysis was used to confirm gene-specific amplification and to rule out the non-specific amplification and the generation of primer-dimer. The standard curve for each candidate was generated from cDNA 10-fold dilution series (1/10, 1/10^2^, 1/10^3^, 1/10^4^, and 1/10^5^). The corresponding RT-qPCR efficiencies (E) were expressed in percentage according to the equation: *E* = (10^[–1/slope]^ − 1) × 100%. A total of three biological replication were carried out for RT-qPCR analysis, with three technical replications for each biological replication. One way ANOVA was carried out to compare the Ct values of each candidate reference gene under different conditions (SAS/STAT^®^ software, SAS Institute, AS Institute Inc., Cary, NC, United States).

### Stability of Candidate Reference Gene Expression

The stability of the candidate HKGs was evaluated by geNorm (Vandesompele et al., 2002), NormFinder (Andersen et al., 2004), BestKeeper (Pfaffl et al., 2004), and the ΔCt method (Silver et al., 2006). Based on geNorm, the gene expression stability was calculated by the *M*-value, and the gene with the lowest M value was considered as the most stable. NormFinder calculates the expression stability value SV for each reference gene. According to BestKeeper, the stability of a gene is directly proportional to the Pearson correlation coefficient (*r*), while it is inversely proportional to the SD value. ΔCt method ranks the stability of candidate reference genes based on the pair-wise comparisons. Using raw Ct value, the average SD of each gene set is inversely proportional to its stability.

The RefFinder^2^ was used for the overall ranking of the candidate reference genes (Xie et al., 2012). RefFinder is a comprehensive systemto integrate the currently available major computational programs (geNorm, Normfinder, BestKeeper, and ΔCt method) to compare and rank the stability of candidate reference genes.

### Validation of Recommended Reference Genes

The reliability of the recommended reference genes was evaluated under the different developmental stages. *CREB-binding protein* (*CBP*, GenBank Accession Number: MN607216), which interacts with SRC and regulates multiple signaling pathways (Janknecht and Hunter, 1996; Yao et al., 1996), was used as the target gene. The RT-qPCR primers of *CBP* were listed in Table 1. The relative expression of *CBP* was assessed by seven normalization factors (NFs), including (1) the most suited, (2) the top two most suited, (3) the top three most suited, (4) the least suited, (5) the top two least suited, (6) the top three least suited, and (7) all the reference genes (as recommended by RefFinder). Relative expression of *CBP* was calculated using the 2^–ΔΔCt^ method (Livak and Schmittgen, 2001). One way ANOVA was used to compare the gene expression level of *CBP* under each normalization treatments. A total of six biological replications were carried out for the validation experiment.

## Results

### Primer Specificity and Efficiency

For each candidate, a single RT-qPCR amplicon was produced and visualized with expected size on a 2% agarose gel. Non-specific bands were not found and a single peak was observed in the melting curve analysis. A standard curve was generated for each gene, using a 10-fold serial dilution of the pooled cDNA. The correlation coefficient (*R*^2^) and PCR efficiency for each standard curve was shown in Table 1. The *R*^2^ ranged between 0.98 and 1.00, and efficiency of RT-qPCR ranged between 90% and 110% (Table 1), which are consistent with previous studies (Taylor et al., 2010).

### Cycle Threshold (Ct) Values

**FIGURE 1:**
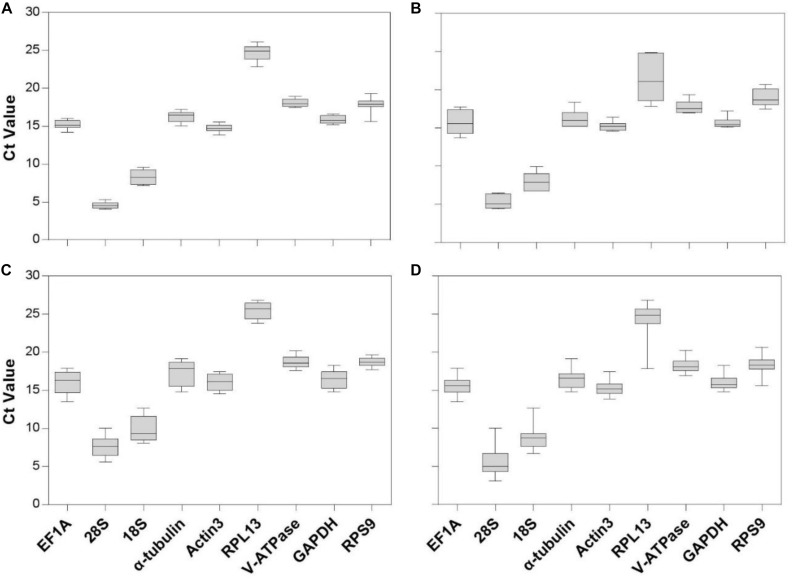
Expression range of Ct values of candidate reference genes under different intrinsic conditions in *A. viennensi*. The intrinsic conditions included different developmental stage **(A)**, sex **(B)**, diapause **(C)**, and all three conditions combined **(D)**. The box indicates the 25th and 75th percentiles, the line across the box represents the median, and whisker caps the maximum and minimum values.

Gene expression analyses of the nine reference genes exhibited a broad range of Ct values, covering all the experimental conditions (Figure 1, Ct values under different conditions were shown in Supplementary Table S1). Ct values ranged from 3.09 to 26.81, while most of the values were distributed between 14 and 19 (Figure 1 and Table 2). *28S* and *18S rRNA* were the most abundant transcripts with the mean Ct values of 5.62 ± 0.34 and 8.76 ± 0.29, and *RPL13 was* the least abundant candidate reference gene (mean Ct value = 24.26 ± 0.44, Table 2). In addition, Ct values of *GAPDH*, *EF1A*, and α*-tubulin* were consistent cross different intrinsic conditions, while other candidates exhibited significant differences (Supplementary Figure S1).

**TABLE 2:** Ct values of candidate reference genes under different intrinsic conditions.

**Candidate gene**	**Developmental stage^∗^**	**Sex**	**Diapause**	**Intrinsic conditions**
*EF1A*	15.21 ± 0.16	15.77 ± 0.69	16.05 ± 0.51	15.61 ± 0.24
*28S*	4.31 ± 0.17	5.32 ± 0.36	7.57 ± 0.48	5.62 ± 0.34
*18S*	8.25 ± 0.24	7.98 ± 0.54	9.97 ± 0.55	8.76 ± 0.29
α*-tubulin*	16.25 ± 0.21	16.23 ± 0.50	17.28 ± 0.55	16.59 ± 0.24
*Actin3*	14.78 ± 0.14	15.23 ± 0.26	16.09 ± 0.37	15.32 ± 0.18
*RPL13*	24.71 ± 0.28	21.43 ± 1.33	25.54 ± 0.37	24.26 ± 0.44
*V-ATPase*	18.05 ± 0.14	17.74 ± 0.36	18.73 ± 0.28	18.21 ± 0.15
*GAPDH*	15.89 ± 0.15	15.67 ± 0.32	16.42 ± 0.39	16.02 ± 0.17
*RPS9*	17.88 ± 0.27	18.95 ± 0.48	18.72 ± 0.20	18.40 ± 0.19

*“^∗^”, Ct values are presented as Ct ± SE.*

### Stability of the Candidate Reference Gene Expression

**FIGURE 2:**
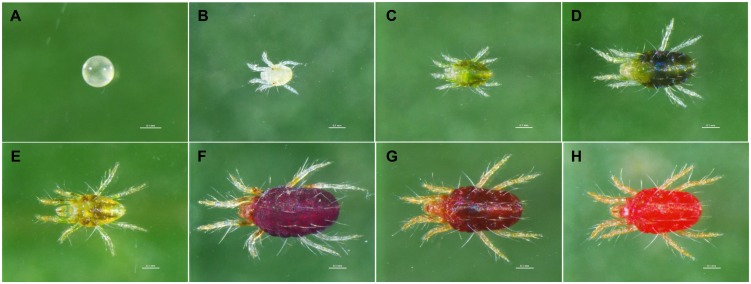
The morphology of *A. viennensis* under different intrinsic conditions. The developmental stages include egg **(A)**, larvae **(B)**, protonymph **(C)**, and deutonymph **(D)**. Sex includes male **(E)** and female **(F)**. Diapause stage includes the normal adult females **(F)**, pre-diapause females **(G)**, and diapause females **(H)**. The scale bar presents 0.1 mm.

The morphological traits of *A. viennensis* under the three intrinsic conditions were shown in Figure 2. The different developmental stages include egg, larvae, protonymph, and deutonymph (Figures 2A–D), which can be easily distinguished by the size and body color. Sex includes male with a milk white or faint yellow body and a smaller size (Figure 2E) and female with a dark red body and a bigger size (Figure 2F). Diapause stage includes a normal female with a dark red body and translucent legs (Figure 2F), a pre-diapause female with a red body, and faint yellow legs (Figure 2G), and a diapause female with a fresh red body and orange legs (Figure 2H).

The stability ranking of the nine candidate genes under the three intrinsic conditions is summarized in Table 3. For different developmental stages of nymphs, the top three most stable candidates were *Actin3*, *V-ATPase*, and *EF1A* based on Normfinder and ΔCt method. GeNorm ranked the top four most stable candidates as *V-ATPase* = *GAPDH*, *Actin3*, and *28S*. While Bestkeeper provided a similar ranking, with *V-ATPase*, *Actin3*, *28S* were rated as the top three candidates. *18S*, the least stable candidate, was not recommended by any computational program. For different sexes, the top three most stable candidates were *Actin3*, *GAPDH*, and *V-ATPase* according to all four computational programs. While *RPL13* was the least stable candidate gene. For diapause, geNorm ranked the stability from high to low as *Actin3*, *V-ATPase*, *GAPDH*, *EF1A*, α*-tubulin*, *RPL13*, *RPS9*, *28S*, *18S*. Normfinder offered a list as follows: *GAPDH*, *Actin3*, *RPL13*, *EF1A*, *V-ATPase*, *RPS9*, α*-tubulin*, and *28S*, *18S*. Bestkeeper provided a ranking as *Actin3* = *GAPDH*, *RPL13*, *V-ATPase*, *EF1A*, *RPS9*, α*-tubulin*, *28S*, and *18S*. ΔCt method ranks from the most stable to the least stable as *Actin3*, *GAPDH*, *RPL13*, *V-ATPase*, *EF1A*, *RPS9*, α*-tubulin*, *28S*, and *18S*.

**TABLE 3:** Stability of candidate reference genes under different intrinsic conditions.

**Conditions**	**Candidate Genes**	**geNorm**		**Normfinder**		**BestKeeper**		**ΔCt method**		**RefFinder**
						
		**Stability**	**Ranking**	**Stability**	**Ranking**	**Stability**	**Ranking**	**Stability**	**Ranking**	
Developmental stages	*EF1A*	0.495	5	0.342	3	0.480	5	0.655	3	*V-ATPase Actin3 GAPDH*
	*28S*	0.425	4	0.587	5	0.426	3	0.771	5	
	*18S*	0.767	9	0.781	9	0.747	8	0.937	9	
	α*-tubulin*	0.566	6	0.596	6	0.614	7	0.788	6	
	*Actin3*	0.369	3	0.049	1	0.392	2	0.584	1	
	*RPL13*	0.658	7	0.721	8	0.777	9	0.889	7	
	*V-ATPase*	0.287	1	0.329	2	0.386	1	0.632	2	
	*GAPDH*	0.287	1	0.537	4	0.455	4	0.726	4	
	*RPS9*	0.718	8	0.750	7	0.585	6	0.917	8	
Sex	*EF1A*	0.744	7	1.294	7	1.412	8	1.520	8	*Actin3 GAPDH V-ATPase*
	*28S*	0.409	4	0.224	4	0.716	4	1.101	4	
	*18S*	0.531	6	0.738	6	1.133	7	1.140	6	
	α*-tubulin*	0.482	5	0.574	5	0.954	6	1.100	5	
	*Actin3*	0.246	1	0.123	1	0.424	1	1.036	2	
	*RPL13*	1.496	9	4.105	9	2.902	9	4.128	9	
	*V-ATPase*	0.271	3	0.128	3	0.643	3	1.008	1	
	*GAPDH*	0.246	1	0.123	1	0.520	2	1.049	3	
	*RPS9*	0.641	8	0.820	7	0.910	5	1.378	7	
Diapause	*EF1A*	0.580	4	0.632	4	0.957	5	0.957	5	*Actin3 GAPDH V-ATPase*
	*28S*	0.912	8	0.945	8	1.203	8	1.203	8	
	*18S*	1.042	9	1.395	9	1.53	9	1.530	9	
	α*-tubulin*	0.659	5	0.860	7	1.096	7	1.096	7	
	*Actin3*	0.478	1	0.469	2	0.857	1	0.857	1	
	*RPL13*	0.751	6	0.487	3	0.942	3	0.942	3	
	*V-ATPase*	0.478	1	0.635	5	0.946	4	0.948	4	
	*GAPDH*	0.503	3	0.439	1	0.857	1	0.858	2	
	*RPS9*	0.808	7	0.744	6	1.051	6	1.051	6	
Intrinsic conditions	*EF1A*	0.670	5	0.797	5	0.978	5	1.200	5	*V-ATPase Actin3 GAPDH*
	*28S*	1.044	8	1.198	8	1.418	8	1.536	8	
	*18S*	0.932	7	0.999	7	1.128	7	1.417	7	
	α*-tubulin*	0.609	4	0.632	4	0.992	6	1.152	4	
	*Actin3*	0.486	3	0.137	1	0.737	4	1.008	1	
	*RPL13*	1.363	9	2.365	9	1.598	9	2.482	9	
	*V-ATPase*	0.366	1	0.389	2	0.648	1	1.052	2	
	*GAPDH*	0.366	1	0.461	3	0.71	2	1.077	3	
	*RPS9*	0.786	6	0.967	6	0.729	3	1.347	6	

Under the selected intrinsic conditions, *18S*, *28S*, and *RPL13* were the least stable candidates. The top three most stable candidate genes were *V-ATPase*, *GAPDH*, and *Actin3* according to geNorm, Normfinder and ΔCt, while Bestkeeper rated *V-ATPase*, *GAPDH* and *RPS9* as the top three most stable reference genes.

### Recommended Reference Genes

**FIGURE 3:**
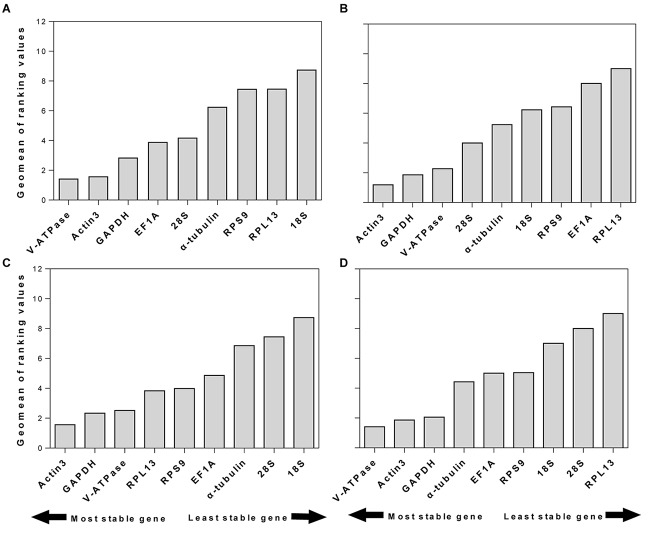
Stability of candidate reference genes under different intrinsic conditions. The intrinsic conditions included different developmental stage **(A)**, sex **(B)**, diapause **(C)**, and all three conditions combined **(D)**. The final stability ranking was provided a comprehensive ranking system, RefFinder, which integrates all four computational platforms used in this study. A lower Geomean value denotes stable expression.

RefFinder is a web-based software, which can generate a comprehensive ranking of reference genes integrating all four above-mentioned programs. The recommended reference genes was listed in Table 3. *V-ATPase*, *Actin3*, and *GAPDH* were the most stable reference genes across different developmental stages (Table 3 and Figure 3A). For different sexes, the top three most stable candidates were *Actin3*, *GAPDH*, and *V-ATPase* (Table 3 and Figure 3B). For diapause, the recommendation was *Actin3, GAPDH*, and *V-ATPase* (Table 3 and Figure 3C). Based on the comprehensive ranking of RefFinder, the most to the least stable candidate reference genes under the intrinsic conditions was: *V-ATPase*, *Actin3*, *GAPDH*, α*-tubulin*, *EF1A*, *RPS9*, *18S*, 28S, and *RPL13* (Figure 3D).

### Optimal Number of Candidate Reference Genes

**FIGURE 4:**
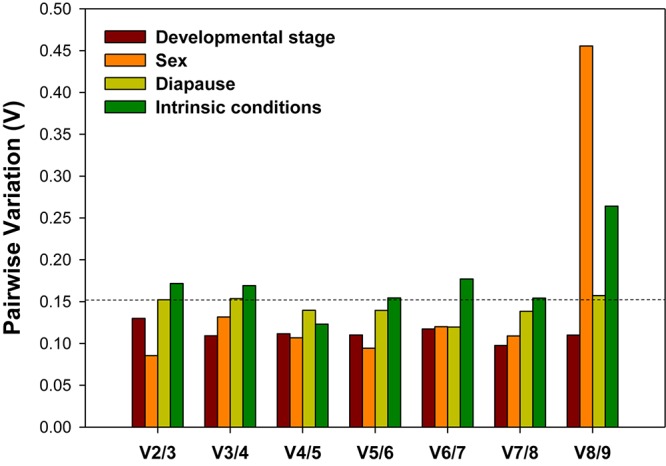
Optimal number of reference genes for the normalization of *A. viennensi* gene expression under different intrinsic conditions. The pairwise variation (Vn/Vn + 1) was analyzed for the normalization factors by geNorm program to determine the optimal number of reference genes included in the RT-qPCR analysis. Values less than 0.15 suggest that the addition of another reference gene will not significantly improve the normalization.

For accurate and consistent analyses, two or more reference genes are required for RT-qPCR analysis. All pairwise variations (Vn/n + 1) under each experimental conditions were evaluated using geNorm. The cutoff value for the optimal number of reference genes for the reliable normalization is 0.15. When Vn/n + 1 < 0.15, it means that the inclusion of an addition reference gene (n + 1) is not necessary. For the developmental stage and sex, the first *V*-value less than 0.15 occurred at V2/3, suggesting that two reference genes were sufficient for normalization (Figure 4). For diapause, although the first *V*-value less than 0.15 appeared at V4/5, indicating that four reference genes were warranted for accurate normalization, we would also consider to use two reference genes due to the fact that V2/3 was approximately 0.15 (Figure 4).

### Validation of Recommended Reference Genes

**FIGURE 5:**
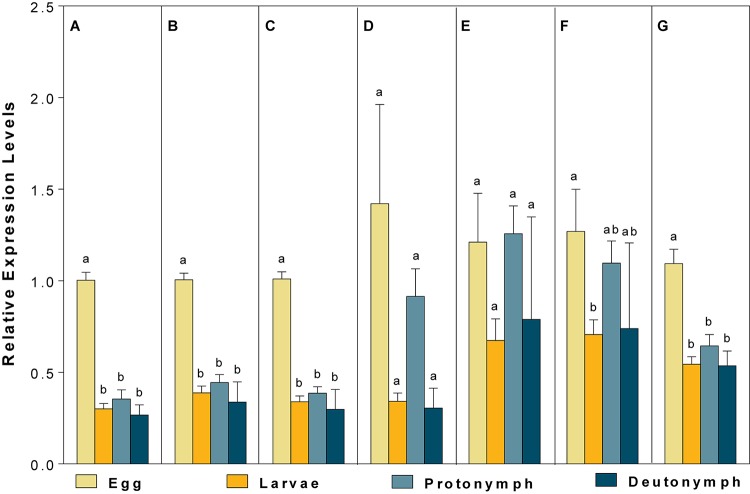
Validation of the recommended reference genes. Expression profiles of *CBP* among different developmental stages were investigated using seven different normalization factors, including **(A)** the most suited (*V-ATPase*), **(B)** the top two most suited (*V-ATPase, Actin3*), **(C)** the top three most suited (*V-ATPase, Actin3, GAPDH*), **(D)** the least suited (*18S*), **(E)** the top two least suited (*18S, RPL13*), **(F)** the top three least suited (*18S*, *RPL13*, *RPS9*), and **(G)** all the candidate reference genes. Bars represent the means ± standard error of six biological replications. Different letters indicate significant differences (*P* < 0.05).

For different developmental stages, *CBP* expression profiles were different when normalized to (1) the most (*V-ATPase*) and least suited (*18S*), (2) the two most (*V-ATPase* and *Actin3*) and least suited (*18S* and *RPL13*), (3) the three most (*ATPase*, *Actin3*, and *GAPDH*) and least suited genes (*18S*, *RPL13* and *RPS9*) (as recommended by RefFinder), and (4) all the candidate reference genes (Figure 5). Specifically, the expression pattern of *CBP*, in which *CBP* expression was significantly elevated in eggs, was consistent when the most stable (*V-ATPase*, Figure 5A), the top two most suited (*V-ATPase and Actin3*, Figure 5B), the top three most suited (*ATPase, Actin3 and GAPDH*, Figure 5C), and all the candidate reference genes (Figure 5G) was used as the normalizer. In comparison, the differential expression pattern of *CBP* among developmental stages was not the statistically evident when the least suited (*18S*, Figure 5D), the top two least suited (*18S and RPL13*, Figure 5E), and the top three least suited reference genes (*18S*, *RPL13*, and *RPS9*, Figure 5F) were used as the normalizer.

## Discussion

Gene expression analyses across different experimental conditions are essential to understand the molecular mechanisms underlying various biological processes (Nolan et al., 2006). As a powerful technology to analyze gene expression, RT-qPCR is superior comparing to other conventional methods because of its accuracy and sensitivity. However, finding the right reference genes is a key to offset the innate variations and biases within the gene expression analysis (Lü et al., 2018). The candidate reference genes, therefore, should be evaluated under specific experimental conditions to minimize RT-qPCR analysis errors (Thellin et al., 1999; Lü et al., 2018).

**FIGURE 6:**
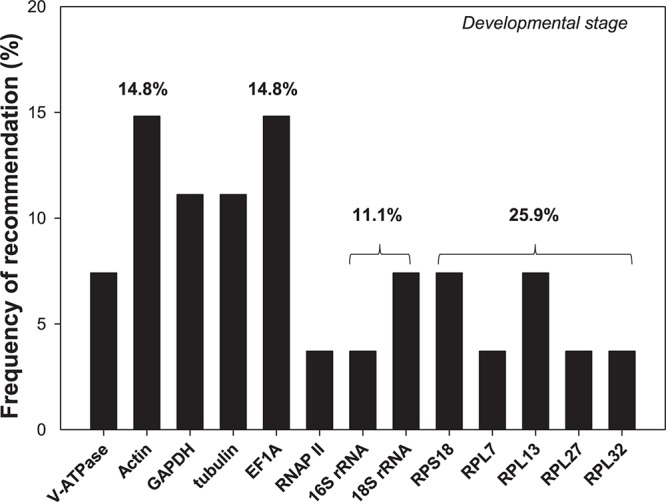
Frequency of reference genes recommended for the cell-content feeding arthropod pests. Here we surveyed the frequency of each reference gene recommended for different developmental stages among the nine cell-content feeding arthropod species, including four spider mites, *A. viennensis, Tetranychus urticae, Tetranychus cinnabarinus*, and *Panonychus citri*, and five hemipterans, *Myzus persicae, Aphis gossypii, Toxoptera citricida, Lipaphis erysimi*, and *Sogatella furcifera*. Reference genes (top three) recommended for each species under different developmental stages are detailed in Supplementary Table S2.

Selection of reference genes has been conducted for an array of cell-content feeding arthropods (Bensoussan et al., 2016, 2018), including three spider mites species, *T. urticae* (Yang et al., 2015a), *Tetranychus cinnabarinu* (Sun et al., 2010), and *Panonychus citri* (Niu et al., 2012), and five Hemiptera species, *Myzus persicae* (Kang et al., 2017), *Aphis gossypii* (Ma et al., 2016), *Toxoptera citricida* (Shang et al., 2015), *Lipaphis erysimi* (Koramutla et al., 2016), and *Sogatella furcifera* (An et al., 2016; Supplementary Table S2). In this study, we added hawthorn spider mites, *A. viennensis*, to this list. Developmental stages is the only experimental condition in common for all four spider mites and five hemipterans. Among the 13 reference genes recommended for the nine cell-content feeding arthropods, two of them were rRNAs (*16S* and *18S*), and five were ribosomal proteins (*RPS18, RPL7, RPL13, RPL27*, and *RPL32*). Based on the frequency of being selected, ribosomal proteins were the top choice (25.9%), in which ribosomal protein S (*RPS18*) accounted for 7.4% chance, while ribosomal protein L (*RPL7, RPL13, RPL27*, and *RPL32*) had a 18.5% probability (Figure 6). Given their abundance in the cell, rRNAs and ribosomal proteins are consistently stably expressed throughout the vast majority of biotic and abiotic conditions, and therefore, they has been adopted by many as the internal references. The over-representation of rRNAs in the total RNAs (>80%), however, can potentially mask the subtle changes of the target gene expression, which negatively affect its utility as the normalizer (Yang et al., 2018). In addition, *EF1A, Actin, GAPDH*, and *tubulin* all exceeded 10% chance of been selected (Figure 6).

Results from this study further validate this observation. Specifically, *V-ATPase* was recommended for the developmental stages, while *Actin3* was the top choice for both sex and diapause in *A. viennensis*. Overall, *V-ATPase* was the most stable reference gene across all the intrinsic conditions, and followed by *Actin3*. *GAPDH*, a highly conserved enzyme in eukaryotic organisms, functions as a proton pump to transport across intracellular and plasma membranes of eukaryotic cells (Nelson et al., 2000). Under different developmental stages, *V-ATPase* was the most stable reference gene in a ladybeetle, *Coleomegilla maculata* (Yang et al., 2015c) and among the top three reference genes for the two-spotted spider mites, *T. urticae* (Yang et al., 2015a). *Actin* belongs to a protein family, which forms microfilaments, and plays an essential role in all eukaryotic cells. *Actin* has been ranked as the most stable reference genes under different developmental stages in *Liriomyza trifolii* and *Diabrotica virgifera* (Rodrigues et al., 2014; Chang et al., 2017). In addition, *Actin* was ranked as the most stable reference gene between sexes for *Galeruca daurica* (Tan et al., 2017). However, this was not the case for *Hippodamia convergens*, *G. daurica*, *C. maculata*, *Sesamia inferens* and *L. trifolii* (Pan et al., 2015; Sun et al., 2015; Yang et al., 2015c; Chang et al., 2017; Tan et al., 2017). In addition, *Actin* was unstable during diapause in *G. daurica* (Tan et al., 2017).

A wealth of information from previous research suggests that a single reference gene will lead to deviations in RT-qPCR analysis, therefore two or more reference genes were necessary to avoid biased normalization (Yang et al., 2018). The optimal number of reference genes is determined by geNorm based on *M-values*. Specially, two reference genes are recommended for the reliable normalization under different developmental stages (*V-ATPase* and *Actin3*) and sex conditions (*Actin3* and *GAPDH*); while four reference genes are sufficient during diapause (*Actin3*, *V-ATPase, GAPDH* and *EF1A*). In addition, our validation study clearly demonstrates the necessity for such research. When using the most stably expressed internal reference(s), the gene expression profile is substantially more accurate than using the least suited reference(s). Although combing all the candidates as the normalizer offers a comparable accuracy, it is practically impossible to accomplish in the real experiment.

This research represents an important first step toward establishing a standardized RT-qPCR analysis procedure for *A. viennensis*. Overall, *V-ATPase*, *Actin3*, *GAPDH* and α*-tubulin* are the recommended reference genes for all there intrinsic conditions (Figure 4). With the sequencing of *A. viennensis* genome and transcriptomes, results from this study lays the foundation for the subsequent genomics and functional genomics research.

## Data Availability Statement

The raw data supporting the conclusions of this manuscript will be made available by the authors, without undue reservation, to any qualified researcher.

## Author Contributions

XZ, RF, and JY designed the experiments. ZL and YG collected the samples from field. JY and YZ carried out the experiments and did the analysis. JY drafted the manuscript. XZ revised the manuscript. All authors read and approved its final version.

## Conflict of Interest

The authors declare that the research was conducted in the absence of any commercial or financial relationships that could be construed as a potential conflict of interest.

## Footnotes

1 http://www.bioinformatics.nl/cgi-bin/primer3plus/primer3plus.cgi/

2 https://www.heartcure.com.au/reffinder/?type=reference
