# Supplementary material for: Selection of Reference Genes for RT-qPCR Analysis Under Intrinsic Conditions in the Hawthorn Spider Mite, Amphitetranychus viennensis (Acarina: Tetranychidae)
Source: Front Physiol. 2019 Nov 19;10:1427. doi: 10.3389/fphys.2019.01427 (PMC6877696; doi:10.3389/fphys.2019.01427)
Supplement: Supplementary file 1 [file Table_1.docx]

**Table S1. All the Ct Values under different experimental conditions.**

| **Intrinsic Conditions** | | ***EF1A*** | ***28S*** | ***18S*** | ***Tubulin*** | ***Actin3*** | ***RPL13*** | ***ATPase*** | ***GAPDH*** | ***RPS9*** |
| --- | --- | --- | --- | --- | --- | --- | --- | --- | --- | --- |
| **Developmental stages** | **egg1** | 14.60 | 4.78 | 7.61 | 15.57 | 14.40 | 23.39 | 18.05 | 16.62 | 15.60 |
|  | **egg2** | 14.16 | 4.29 | 7.79 | 15.05 | 13.84 | 22.84 | 17.75 | 15.64 | 18.23 |
|  | **egg3** | 15.27 | 4.99 | 8.08 | 16.01 | 15.13 | 23.74 | 18.81 | 16.61 | 17.47 |
|  | **larva1** | 14.99 | 3.09 | 7.17 | 16.39 | 14.66 | 24.22 | 17.59 | 15.29 | 17.79 |
|  | **larva2** | 16.04 | 5.32 | 9.32 | 16.79 | 15.51 | 25.68 | 18.73 | 16.36 | 19.27 |
|  | **larva3** | 15.82 | 4.18 | 7.66 | 16.63 | 14.74 | 24.79 | 17.85 | 15.79 | 17.92 |
|  | **protonymph 1** | 14.89 | 4.13 | 9.54 | 15.64 | 14.43 | 24.86 | 17.48 | 15.17 | 17.22 |
|  | **protonymph 2** | 14.76 | 4.31 | 9.12 | 15.29 | 14.68 | 24.97 | 17.58 | 15.51 | 17.79 |
|  | **protonymph 3** | 15.77 | 4.36 | 8.84 | 16.72 | 15.54 | 26.11 | 18.93 | 16.44 | 19.00 |
|  | **Deutonymph 1** | 15.61 | 3.58 | 7.19 | 17.20 | 14.34 | 24.98 | 17.83 | 15.37 | 17.88 |
|  | **Deutonymph 2** | 14.97 | 4.66 | 8.89 | 16.76 | 15.08 | 25.49 | 18.06 | 16.17 | 18.05 |
|  | **Deutonymph 3** | 15.61 | 4.03 | 7.74 | 16.94 | 14.98 | 25.48 | 17.97 | 15.74 | 18.31 |
| **Sexes** | **male1** | 16.20 | 5.27 | 8.71 | 16.60 | 15.21 | 18.81 | 18.05 | 15.53 | 20.66 |
|  | **male2** | 17.63 | 6.32 | 8.68 | 16.59 | 15.33 | 17.85 | 17.59 | 15.59 | 18.62 |
|  | **male3** | 17.72 | 6.47 | 9.94 | 18.36 | 16.41 | 18.93 | 19.37 | 17.23 | 19.98 |
|  | **female1** | 14.44 | 4.38 | 6.73 | 15.23 | 14.68 | 24.90 | 17.09 | 15.25 | 17.47 |
|  | **female2** | 14.95 | 4.92 | 7.09 | 15.21 | 15.18 | 24.79 | 17.44 | 15.33 | 18.76 |
|  | **female3** | 13.68 | 4.55 | 6.72 | 15.39 | 14.59 | 23.31 | 16.93 | 15.10 | 18.22 |
| **Diapause stages** | **diapause1** | 17.08 | 6.72 | 9.31 | 18.35 | 16.76 | 25.61 | 19.68 | 17.23 | 18.62 |
|  | **diapause2** | 17.92 | 7.99 | 10.33 | 19.16 | 17.47 | 26.15 | 20.21 | 18.27 | 18.72 |
|  | **diapause3** | 17.75 | 9.34 | 11.50 | 18.65 | 16.56 | 25.74 | 18.94 | 17.66 | 19.38 |
|  | **pre-diapause1** | 15.73 | 7.83 | 12.66 | 17.84 | 15.84 | 26.55 | 18.39 | 15.99 | 18.49 |
|  | **pre-diapause2** | 16.32 | 10.04 | 11.70 | 16.84 | 16.17 | 26.81 | 18.56 | 16.56 | 19.04 |
|  | **pre-diapause3** | 16.78 | 7.67 | 8.47 | 18.78 | 17.46 | 26.44 | 19.07 | 16.65 | 19.68 |
|  | **Non-diapause 1** | 14.49 | 6.22 | 8.46 | 14.78 | 14.56 | 24.11 | 17.60 | 15.03 | 18.05 |
|  | **Non-diapause 2** | 13.51 | 5.56 | 8.09 | 15.32 | 14.59 | 23.82 | 17.74 | 14.80 | 17.72 |
|  | **Non-diapause 3** | 14.89 | 6.74 | 9.21 | 15.77 | 15.38 | 24.61 | 18.37 | 15.57 | 18.83 |
