# Supplementary material for: Selection of Reference Genes for RT-qPCR Analysis Under Intrinsic Conditions in the Hawthorn Spider Mite, Amphitetranychus viennensis (Acarina: Tetranychidae)
Source: Front Physiol. 2019 Nov 19;10:1427. doi: 10.3389/fphys.2019.01427 (PMC6877696; doi:10.3389/fphys.2019.01427)
Supplement: Supplementary file 2 [file Table_2.docx]

**Table S2. Recommended reference genes for RT-qPCR analysis under different developmental stages in cell-content feeding pests**

| **Species** | **Candidate Genes** | **Recommendation** | **Program** | **Reference** |
| --- | --- | --- | --- | --- |
|  | **Tetranychidae** | | | |
| *Amphitetranychus viennensis* | *18S*, *28S*, *EF1A*, *Actin3*, *V-ATPase*, α*-tubulin*, *RPL13*, *RPS9*, *GAPDH* | *V-ATPase*, *Actin3*, *GAPDH* | RefFinder | This study |
| *Tetranychus urticae* | *18S*, *28S*, *EF1A*, *V-ATPase*, *SDHA*, *GAPDH*, α*-tubulin*, *RPL13*, *RP49*, *Actin* | *RPL13*, *GAPDH*, *V-ATPase* | RefFinder | Yang et al., 2015a |
| *Tetranychus cinnabarinus* | *5.8S*, α*-tubulin*, *Actin*, *GAPDH*, *RPL13a*, *RPS18*, *SDHA*, *TBP* | *RPS18*, α*-tubulin*, *RPL13a* | GeNorm and NormFinder | Sun et al., 2010 |
| *Panonychus citri* | *Actin*, α*-tubulin*, *EF1A*, *GAPDH*, *RNAP II*, *SDHA*, *5.8S* | *EF1A*, *RNAP II*, α*-tubulin* | GeNorm | Niu et al., 2012 |
|  | **Hemiptera** | | | |
| *Myzus persicae* | *Actin*, *RPL27*, *RPL7*, *β-tubulin*, *GAPDH*, *ACE*, *18S*, *EF-1A*, *RPL32* | *RPL27*, *18S*, *RPL32* | RefFinder | Kang et al., 2017 |
| *Aphis gossypii* | *18S*, *28S*, *Actin*, *GAPDH*, *EF1A*, *RPL7*, α*-tubulin*, *TBP* | *EF1A*, *Actin*, *RPL7* | RefFinder | Ma et al., 2016 |
| *Toxoptera citricida* | *EF1A*, *18S*, *RNAP II*, *Actin*, α*-tubulin*, *GAPDH* | *EF1A*, *18S*, *Actin* | RefFinder | Shang et al., 2015 |
| *Lipaphis erysimi* | *16S*, *SDHB*, *Actin*, *EF1A*, *RPL13*, *RPS18*, *RPL27*, *RPL29*, *β-tubulin*, *GAPDH* | *16S*, *GAPDH*, *RPS18* | GeNorm | Koramutla et al., 2016 |
| *Sogatella furcifera* | *EF1A*, *UB*, *RPS18*, *Actin1*, α*-tubulin*, *GAPDH*, *RPL9*, *RPL10*, *18S* | α*-tubulin*, *EF1A*, *Actin1* | RefFinder | An et al., 2016 |
