# Supplementary material for: Selection of Reference Genes for RT-qPCR Analysis Under Intrinsic Conditions in the Hawthorn Spider Mite, Amphitetranychus viennensis (Acarina: Tetranychidae)
Source: Front Physiol. 2019 Nov 19;10:1427. doi: 10.3389/fphys.2019.01427 (PMC6877696; doi:10.3389/fphys.2019.01427)
Supplement: Supplementary file 3 [file Image_1.pdf]

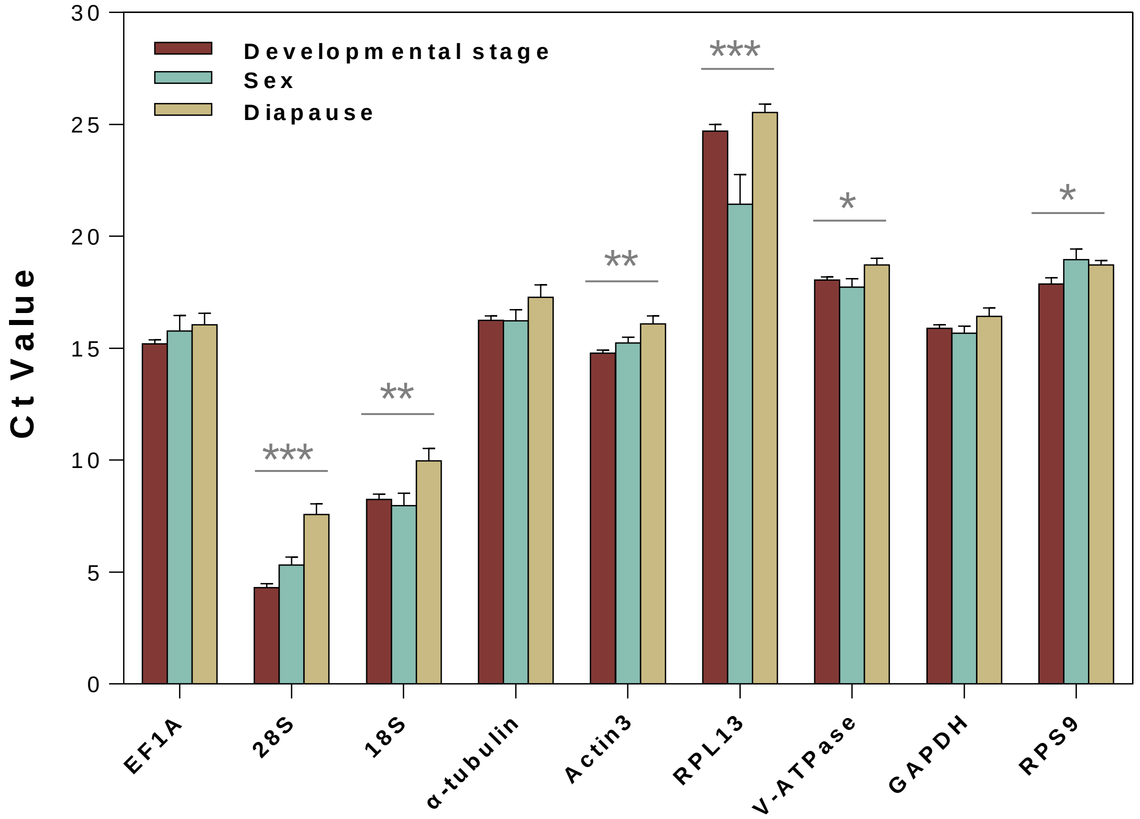

Figure S1. Statistical analysis of Ct values for each candidate reference gene across different intrinsic conditions in *A. viennensi*. Bars represent the means ± standard error of three biological replications. * denotes significant differences among three intrinsic conditions (* $P < 0.05$, ** $P < 0.01$, *** $P < 0.001$).
